# An epidemiological investigation of a Forkhead box protein E3 founder mutation underlying the high frequency of sclerocornea, aphakia, and microphthalmia in a Mexican village

**Published:** 2013-08-27

**Authors:** Carlos Pantoja-Melendez, Manir Ali, Juan C. Zenteno

**Affiliations:** 1 Research Unit-Genetics, Institute of Ophthalmology, “Conde de Valenciana,” Mexico City, Mexico; 2 Department of Public Health, Faculty of Medicine, National Autonomous University of Mexico, Mexico City, Mexico; 3 Leeds Institute of Molecular Medicine, University of Leeds, Leeds, United Kingdom; 4 Department of Biochemistry, Faculty of Medicine, National Autonomous University of Mexico, Mexico City, Mexico

## Abstract

**Purpose:**

To investigate the molecular epidemiological basis for the unusually high incidence of sclerocornea, aphakia, and microphthalmia in a village in the Tlaxcala province of central Mexico.

**Methods:**

A population census was performed in a village to identify all sclerocornea, aphakia, and microphthalmia cases. Molecular analysis of the previously identified Forkhead box protein E3 (*FOXE3*) mutation, c.292T>C (p.Y98H), was performed with PCR amplification and direct DNA sequencing. In addition, DNA from 405 randomly selected unaffected villagers was analyzed to establish the carrier frequency of the causal mutation. To identify the number of generations since the mutation arose in the village, 17 polymorphic markers distributed in a region of 6 Mb around the mutated locus were genotyped in the affected individuals, followed by DMLE software analysis to calculate mutation age.

**Results:**

A total of 22 patients with sclerocornea, aphakia, and microphthalmia were identified in the village, rendering a disease prevalence of 2.52 cases per 1,000 habitants (1 in 397). The *FOXE3* homozygous mutation was identified in all 17 affected subjects who consented to molecular analysis. Haplotype analysis indicated that the mutation arose 5.0–6.5 generations ago (approximately 106–138 years). Among the 405 unaffected villagers who were genotyped, ten heterozygote carriers were identified, yielding a population carrier frequency of approximately 1 in 40 and a predicted incidence of affected of 1 in 6,400 based on random marriages between two carriers in the village.

**Conclusions:**

This study demonstrates that a cluster of patients with sclerocornea, aphakia, and microphthalmia in a small Mexican village is due to a FOXE3 p.Y98H founder mutation that arose in the village just over a century ago at a time when a population migrated from a nearby village because of land disputes. The actual disease incidence is higher than the calculated predicted value and suggests non-random marriages (i.e., consanguinity) within the population. We can now offer the community more informed genetic counseling based on an accurate genetic test, thus increasing the likelihood of a better outcome for the families.

## Introduction

Mexican mestizos are a recently admixed population composed of Amerindian, European, and, to a lesser extent, African ancestry. Before the Spanish arrived in 1519, Mexico was occupied by a large number of Indian groups with different social and economic systems. There was greater diversity and more indigenous Amerindian groups lived in Central and Southern Mexico compared to the northern regions. Between 1545 and 1548, the Amerindian population notably decreased, due to catastrophic epidemics that killed an estimated 80% of the native population [1]. African slaves were brought into the regions soon after due to the labor shortage, and subsequent admixture processes in geographically distant regions have been affected by different demographic and historical conditions, shaping the modern genomic structure of Mexicans. These factors initially generated considerable genetic heterogeneity between subpopulations from different regions throughout Mexico [2]. However, with subsequent generations and the practice of consanguineous marriages, which is still common in several communities, the genetic variation in these isolated populations decreases, thus increasing the chances of recessive disease. For example, when a rare disease arises frequently in a population where there is consanguinity, the disease is more likely to arise due to a mutation derived from a common ancestor and is identical-by-descent (IBD), when compared to a population where there is no obvious consanguinity [3].

Sclerocornea is a non-progressive, non-inflammatory uncommon anterior segment dysgenesis (ASD) disorder in which the normal scleral tissue extends beyond the limbus into the peripheral cornea, causing opacification and vascularization [4]. Sclerocornea is typically a bilateral anomaly and can present as total opacification of the cornea (sclerocornea totalis) or as a mild corneal defect characterized by peripheral corneal vascularization (scleralization). Sclerocornea has been proposed to arise from a disrupted migration of neural crest cells between the corneal epithelium and the endothelium during fetal development [5,6]. The majority of cases occur sporadically, but recessive and dominant familial inheritance is also well documented [7,8]. Sclerocornea can occur alone, in association with other ocular defects, or with systemic features as part of a known syndromic entity [9,10].

Recently, we described a consanguineous Mexican pedigree who presented with congenital, non-syndromic, bilateral, total sclerocornea, aphakia, microphthalmia, and optic disc coloboma [11]. Molecular investigations identified a novel Forkhead box protein E3 (*FOXE3*) homozygous missense mutation, c.292T>C, p.Y98H, as the cause of this unusual ocular phenotype in that kindred, which originated from an isolated village in the central part of Mexico. In the current study, we identified additional cases of sclerocornea, aphakia, and microphthalmia in that village and performed a molecular epidemiologic study to screen a large unselected sample of the residents to establish the carrier frequency of the *FOXE3* p.Y98H mutation. Additionally, haplotypes linked to the mutation were characterized to estimate the time since the *FOXE3* mutation arose in this isolated community. Our study offers another example of genetic isolation combined with consanguinity causing a high prevalence of an uncommon ophthalmic disease.

## Methods

### Patient recruitment

The study was performed in a village located in the Tlaxcala state, in central Mexico. One of the authors (C P-M), a clinician with good knowledge about the community, performed a population census by visiting all individual households, interviewing household members to obtain information regarding visual deficiency and/or eye malformations, and collecting samples with the consent of the donors. The investigation was approved by the Institutional Review Board, and all patient samples were collected with written informed consent. The screening program including genetic counseling, and testing was provided free of charge, financed by the Institute of Ophthalmology Conde de Valenciana, in Mexico City.

### Clinical examination of affected individuals

Ophthalmological evaluation was performed in all identified patients. Ocular ultrasonography (modes A and B) was performed in a subgroup of affected individuals.

### Forkhead box protein E3 mutation analysis

Genomic DNA was isolated from buccal cells using the Gentra Puregene buccal cell kit (Qiagen, Valencia, CA) following the manufacturer’s recommendations. To screen for the previously identified c.292T>C, p.Y98H *FOXE3* mutation [11], polymerase chain reaction (PCR)-based direct sequencing was performed using primers 5′-GGG GCC GTG TCC ATA TAA AG-3′ (forward) and 5′-GTT CGA CAA CGG CAG CTT-3′ (reverse), to amplify a gene fragment including *FOXE3* codon 98. Each 25 μl PCR contained 1X buffer, 200 ng of genomic DNA, 0.2 mM of each deoxynucleotide triphosphate, 2U Taq polymerase, 1 mM of forward and reverse primers, and 1.5 mM MgCl_2_. Amplified templates were purified using the Qiaex II kit (Qiagen, Hilden, Germany). Direct automated sequencing of *FOXE3* was performed with the BigDye Terminator Cycle Sequencing kit (Applied Biosystems, Foster City, CA) on an ABI Prism 3130 Genetic Analyzer (Applied Biosystems).

### Estimation of the mutation carrier frequency

The carrier frequency of the *FOXE3* c.292T>C mutation was established by analyzing DNA from 405 randomly selected inhabitants of the village. This sample size was calculated following the formula for an infinite population (95% confidence, 80% of power). As the carrier frequency was unknown, a 0.5 value was used for this calculation. Genotyping was performed using direct nucleotide sequencing, as described above.

### Estimation of mutation age

**Figure 1:**
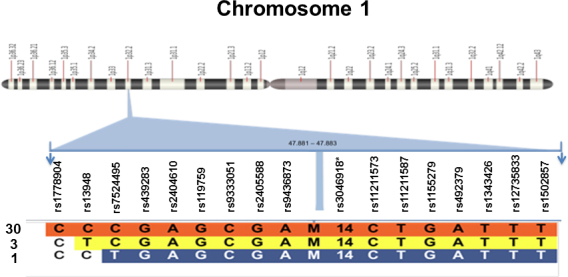
Schematic diagram of the position of 17 polymorphic markers at 1p32 used for haplotype construction in DNA from 17 subjects (34 alleles) with sclerocornea, aphakia, and microphthalmia. The name of the marker and nucleotide genotype are indicated. Three distinct haplotypes were observed in 30 (orange), three (yellow), and one (blue) allele, respectively. Note that rs3046918 (asterisk) is a microsatellite marker. M indicates the mutation locus.

To calculate the time elapsed since the mutation arose in the community, polymorphic markers located 3 Mb on each side of the *FOXE3* locus (1p32) were genotyped to construct the haplotypes linked to the c.292T>C mutation. DMLE+ version 2.2 developed by Reeve and Rannala [12] was used to estimate the age of the *FOXE3* p.Y98H mutation. This program was designed to map a disease mutation in high resolution and estimate its age. The method is based on the observed linkage disequilibrium between a disease mutation and linked markers in DNA samples of affected patients. The program uses the Markov Chain Monte Carlo algorithm for Bayesian estimation of the mutation age [12]. For the population growth rate, we took data from the Mexican Institute of Statistics and Geography (INEGI). Two extreme growth rates were considered: The highest growth rate recorded was 0.65, and the lowest growth rate was 0.5. Genotyped markers included 16 single nucleotide polymorphisms and one microsatellite (Figure 1) and were selected from the December 2011 UCSC browser assembly [13].

## Results

### Actual prevalence of sclerocornea, aphakia, and microphthalmia in a Mexican village

The village population census showed that the community was populated by 8,732 individuals from the Nahuatl indigenous group and the mestizo population. From individual household interviews, a total of 22 patients with sclerocornea, aphakia, and microphthalmia were identified. Thus, a disease prevalence of 2.52 cases per 1,000 habitants, that is, 1 in 397, was established. The 22 affected subjects were distributed among six individual pedigrees (including the pedigree we previously reported [11]).

**Figure 2:**
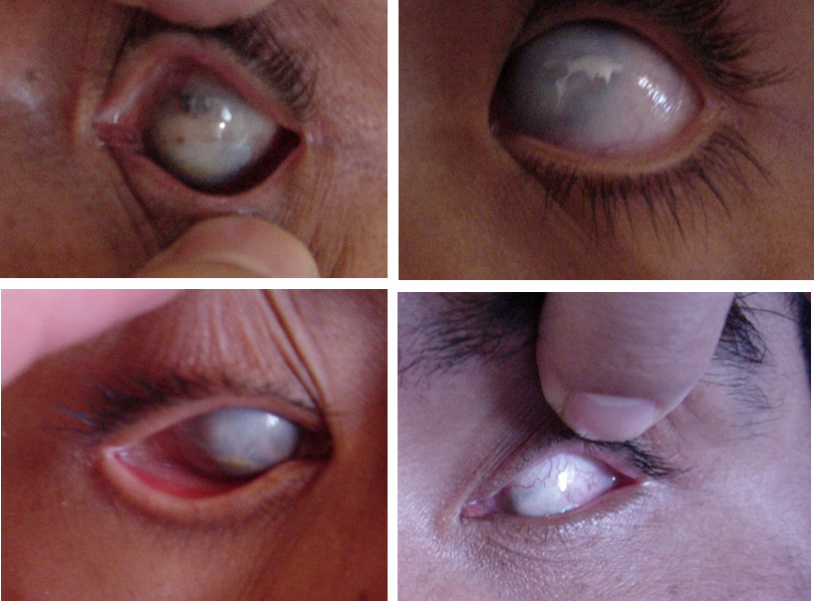
Phenotypic appearance in patients with sclerocornea, aphakia, and microphthalmia. Sclerocornea *totalis* is evident. Visual acuity in affected patients ranged from no light perception to hand movements.

All affected patients presented a uniform bilateral ocular malformation characterized by the absence of identifiable corneal structures with no anatomic limbal delimitation between the cornea and the sclera (Figure 2). None of the patients had extraocular malformations or intellectual disabilities. The ophthalmologic examination revealed a flat cornea, the absence of the anterior chamber of the eye, keratomalacia, ectropion, and increased intraocular pressure. Ocular ultrasonography in seven affected adult subjects revealed a decrease in the length of the anteroposterior axis of the eyes (bilateral microphthalmia), bilateral absence of the crystalline (congenital aphakia), and bilateral coloboma of the optic disc. Visual acuities ranged from no light perception to hand motions. Visual deficiency was congenital in all patients.

Molecular analysis confirmed homozygosity for the *FOXE3* c.292 T>C mutation (p.Y98H) in all 17 cases available for testing. Five affected patients refused to be molecularly screened. The analysis of DNA from the healthy parents of the 17 patients showed the mutation in a heterozygous state.

### Frequency of mutation carriers

To identify the prevalence of mutation carriers in a random sample of people from the community, genomic DNA from 405 healthy individuals who were born in the village was screened for the *FOXE3* mutation. Ten individuals (2.5%) carrying the c.292T>C (p.Y98H) mutation were identified. According to these observations, there is a heterozygote carrier for every 40 residents in the village. Thus, the theoretical probability of a marriage between two carriers is 1 in 1,600 marriages, and the birth of affected children based on random mating is predicted to be 1 in 6,400. The actual value for the number of sclerocornea, aphakia, and microphthalmia cases in the village was calculated as 1 in 397, a higher prevalence than the predicted value due to the practice of consanguinity in the village.

### Estimating the age of the Forkhead box protein E3 c.292T>C, p.Y98H, mutation

For estimating the mutation age, genomic DNA from the 17 confirmed cases (34 alleles) with sclerocornea, aphakia, and microphthalmia were genotyped with 17 polymorphic markers located 3 Mb on either side of *FOXE3*. Three distinct haplotypes were identified: The most common haplotype, and thus considered the ancestral one, was observed in 30 out of 34 alleles; a second haplotype was identified in three alleles while a third haplotype was present in the remaining allele (Figure 1). The calculation obtained using the DMLE program (version 2.2) with a generation time of 21 years was 5.0–6.5 generations, which corresponded to a range of 106–138 years.

## Discussion

In a previous report, we described a Mexican pedigree segregating an autosomal recessive ocular malformation characterized by bilateral sclerocornea, aphakia, and microphthalmia, caused by a *FOXE3* p.Y98H mutation [11]. In the present study, we observed a relatively high prevalence of this anomaly with a prevalence of 1 in 397 in an isolated village. This figure is in sharp contrast with the reported prevalence for the entire group of corneal opacities which is about 3 per 100,000 live births [14].

We also identified the carrier frequency of this causal mutation in the community and predicted that based on random marriages the frequency of affected ought to be 1 in 6,400. These data indicate that non-random marriages (consanguinity) accounts for the elevated frequency of the disease in the community, which is consistent with a recent calculation of an inbreeding coefficient of 0.825 for this population [15]. Consanguineous marriages in this population have been traditionally performed as a way of maintaining land possession among familial clans (personal communications).

*FOXE3* mutations have been previously demonstrated as the cause of sclerocornea and other anterior segment anomalies in humans presenting as a recessive or a dominant condition [16-19]. However, in our study, only individuals with biallelic *FOXE3* mutations gave rise to an ocular phenotype. *FOXE3* mutation carriers within the population, as well as parents of the affected cases, all presented as healthy individuals suggesting that genotype-phenotype correlations, or the genetic background, may account for the differences in inheritance patterns.

The *FOXE3* founder mutation presented in the current report arose more than a century ago. This amount of time coincides with a migration wave in the late 1800s from a neighboring village (Santa Ana) located less than 10 km, in the context of conflicts over land distribution. Interestingly, although all ten mutation carriers and their parents reported they had been born in the village, 55% reported that their grandparents were from the neighboring municipality of Santa Ana. Thus, our data suggest that the “founder” subject arrived approximately 13 decades ago, coming from that neighboring village. A formal search for patients with sclerocornea, aphakia, and microphthalmia was not conducted in the Santa Ana village, but affected individuals could exist there, and the mutation prevalence could be high.

To conclude, our study shows that a cluster of patients with sclerocornea, aphakia, and microphthalmia in a small village from central Mexico was caused by the p.Y98H founder mutation in *FOXE3* and highlights another example of genetic isolation followed by consanguinity causing a high prevalence of an uncommon ophthalmic disease. Consanguineous marriages have been also previously shown to be responsible for the elevated frequency of other autosomal recessive eye diseases in certain populations, as oculocutaneous albinism [20], primary congenital glaucoma [21], Bardet-Biedl syndrome [22], retinitis pigmentosa [23,24], and Hermansky-Pudlak syndrome [25], among others [26].

Haplotype analysis and migration history helped us to trace the probable “age” and origin of this mutation. Our findings have implications for more rational genetic counseling in this community.

## References

[1] Gerhard P. Historical Geography of New Spain, 1519–1821. Mexico City: Universidad Nacional Autonoma de Mexico; 1986. (Spanish)

[2] Silva-Zolezzi I, Hidalgo-Miranda A, Estrada-Gil J, Fernandez-Lopez JC, Uribe-Figueroa L, Contreras A, Balam-Ortiz E, del Bosque-Plata L, Velazquez-Fernandez D, Lara C, Goya R, Hernandez-Lemus E, Davila C, Barrientos E, March S, Jimenez-Sanchez G (2009). Analysis of genomic diversity in Mexican Mestizo populations to develop genomic medicine in Mexico.. Proc Natl Acad Sci USA.

[3] Petukhova L, Shimomura Y, Wajid M, Gorroochurn P, Hodge SE, Christiano AM (2009). The effect of inbreeding on the distribution of compound heterozygotes: a lesson from Lipase H mutations in autosomal recessive woolly hair/hypotrichosis.. Hum Hered.

[4] Elliott JH, Feman SS, O'Day DM, Garber M (1985). Hereditary sclerocornea.. Arch Ophthalmol.

[5] Bahn CF, Falls HF, Varley GA, Meyer RF, Edelhauser HF, Bourne WM (1984). Classification of corneal endothelial disorders based on neural crest origin.. Ophthalmology.

[6] Beauchamp GR, Knepper PA (1984). Role of the neural crest in anterior segment development and disease.. J Pediatr Ophthalmol Strabismus.

[7] Bloch N (1965). Les differents types de sclerocornee, leurs modes d'heredite et les malformations congenitales concomitantes.. J Genet Hum.

[8] Barsky D, Dunn SP (1985). Hereditary sclerocornea.. Henry Ford Hosp Med J.

[9] Idrees F, Vaideanu D, Fraser SG, Sowden JC, Khaw PT (2006). A review of anterior segment dysgeneses.. Surv Ophthalmol.

[10] Mataftsi A, Islam L, Kelberman D, Sowden JC, Nischal KK (2011). Chromosome abnormalities and the genetics of congenital corneal opacification.. Mol Vis.

[11] Ali M, Buentello-Volante B, McKibbin M, Rocha-Medina JA, Fernandez-Fuentes N, Koga-Nakamura W, Ashiq A, Khan K, Booth AP, Williams G, Raashid Y, Jafri H, Rice A, Inglehearn CF, Zenteno JC (2010). Homozygous FOXE3 mutations cause non-syndromic, bilateral, total sclerocornea, aphakia, microphthalmia and optic disc coloboma.. Mol Vis.

[12] Reeve JP, Rannala B (2002). DMLE+: Bayesian linkage disequilibrium gene mapping.. Bioinformatics.

[13] Kent WJ, Sugnet CW, Furey TS, Roskin KM, Pringle TH, Zahler AM, Haussler D (2002). The human genome browser at UCSC.. Genome Res.

[14] Townsend WM. Congenital anomalies of the cornea. In: Kaufman HE, Barron BA, Mc Donald MB, eds. The cornea, 2nd ed, London: Butterworth-Heinemann, 1999. 364-389.

[15] Pierce B. Population Genetics. In: Genetics: a conceptual approach. Chapter 25. W.H. Freeman and Company. New York City. 4th Edition 2012; pp. 693–720.

[16] Valleix S, Niel F, Nedelec B, Algros MP, Schwartz C, Delbosc B, Delpech M, Kantelip B (2006). Homozygous nonsense mutation in the FOXE3 gene as a cause of congenital primary aphakia in humans.. Am J Hum Genet.

[17] Iseri SU, Osborne RJ, Farrall M, Wyatt AW, Mirza G, Nürnberg G, Kluck C, Herbert H, Martin A, Hussain MS, Collin JR, Lathrop M, Nürnberg P, Ragoussis J, Ragge NK (2009). Seeing clearly: the dominant and recessive nature of FOXE3 in eye developmental anomalies.. Hum Mutat.

[18] Brémond-Gignac D, Bitoun P, Reis LM, Copin H, Murray JC, Semina EV (2010). Identification of dominant FOXE3 and PAX6 mutations in patients with congenital cataract and aniridia.. Mol Vis.

[19] Doucette L, Green J, Fernandez B, Johnson GJ, Parfrey P, Young TL (2011). A novel, non-stop mutation in FOXE3 causes an autosomal dominant form of variable anterior segment dysgenesis including Peters anomaly.. Eur J Hum Genet.

[20] Chaki M, Mukhopadhyay A, Chatterjee S, Das M, Samanta S, Ray K (2005). Higher prevalence of OCA1 in an ethnic group of eastern India is due to a founder mutation in the tyrosinase gene.. Mol Vis.

[21] Plásilová M, Stoilov I, Sarfarazi M, Kádasi L, Feráková E, Ferák V (1999). Identification of a single ancestral CYP1B1 mutation in Slovak Gypsies (Roms) affected with primary congenital glaucoma.. J Med Genet.

[22] Hjortshøj TD, Grønskov K, Brøndum-Nielsen K, Rosenberg T (2009). A novel founder BBS1 mutation explains a unique high prevalence of Bardet-Biedl syndrome in the Faroe Islands.. Br J Ophthalmol.

[23] Nevet MJ, Shalev SA, Zlotogora J, Mazzawi N, Ben-Yosef T (2010). Identification of a prevalent founder mutation in an Israeli Muslim Arab village confirms the role of PRCD in the aetiology of retinitis pigmentosa in humans.. J Med Genet.

[24] Ostergaard E, Duno M, Batbayli M, Vilhelmsen K, Rosenberg T (2011). A novel MERTK deletion is a common founder mutation in the Faroe Islands and is responsible for a high proportion of retinitis pigmentosa cases.. Mol Vis.

[25] Anikster Y, Huizing M, White J, Shevchenko YO, Fitzpatrick DL, Touchman JW, Compton JG, Bale SJ, Swank RT, Gahl WA, Toro JR (2001). Mutation of a new gene causes a unique form of Hermansky-Pudlak syndrome in a genetic isolate of central Puerto Rico.. Nat Genet.

[26] Sherwin JC, Hewitt AW, Ruddle JB, Mackey DA (2008). Genetic isolates in ophthalmic diseases.. Ophthalmic Genet.

